# Application of Integrated Fixed-Film Activated Sludge in a Conventional Wastewater Treatment Plant

**DOI:** 10.3390/ijerph19105985

**Published:** 2022-05-14

**Authors:** Magdalena Kuśnierz, Magdalena Domańska, Kamila Hamal, Agnieszka Pera

**Affiliations:** Institute of Environmental Engineering, Faculty of Environmental Engineering and Geodesy, Wrocław University of Environmental and Life Sciences, Plac Grunwaldzki 24, 50-363 Wrocław, Poland; magdalena.kusnierz@upwr.edu.pl (M.K.); kamila.hamal@upwr.edu.pl (K.H.); 113002@student.upwr.edu.pl (A.P.)

**Keywords:** textile fixed bed, IFAS, MMBR, wastewater treatment, FISH, *Stentor* sp.

## Abstract

It is often only at the operation stage of a wastewater treatment plant that there is a need to adjust the treatment process in terms of variable hydraulic capacity, increased pollutant load, high/low concentration of suspended biomass, or the unfavorable phenomenon of reduced sedimentation capacity of the activated sludge. One of the ways to improve the treatment process efficiency is to increase the biologically active surface by using bio-carriers in the form of fibers, materials, or bio-balls. This paper presents the results of a wastewater treatment plant operation during the period of six months after the implementation of the integrated fixed-film activated sludge (IFAS) technology. The research showed that microorganisms developed both in the activated sludge and on the fibers, positively influencing the activated sludge condition. During the start-up of the IFAS process, ciliates predominated over the other species. However, as oxygen content was high (2 mg/dm^3^ and more) and textile beds were used, the protozoan population developed intensively, and small metazoans became increasingly common. Throughout the research period, nitrifying and phosphorus-accumulating bacteria were observed both in the activated sludge and on the fibers. Between the 59th and 184th day of operation, numerous microorganisms were detected on the fibers and in the activated sludge, testifying to low biological oxygen demand, good aerobic conditions for nitrification, and long sludge age. However, the process seemed to break down after day 72, when the occurrence of metazoan led to reduced sludge production; after day 88, chemical oxygen demand and total suspended solids in the outflow increased, and oligochaetes and rotifers dominated the suspended sludge and fibers. Results also showed that the textile bed and low ammonia concentration became an excellent substrate for the development of *Stentor* sp. With regard to chemical and biological oxygen demand, total nitrogen- and total phosphorus-effluent concentrations were mostly within the legally permissible limits throughout the 184 days of operation.

## 1. Introduction

Although the range of technical and technological solutions available at the stage of planning an effective wastewater treatment process is relatively large, a number of unforeseen problems often appear already at the operational stage. There are a lot of wastewater treatment plants (WWTPs) whose operators struggle with variable hydraulic performance [1], an increased inflow of the pollutant load [2,3], high/low concentration of suspended biomass [4,5,6], and the unfavorable phenomenon of lowered sedimentation capacity of activated sludge [7,8,9]. The causes of such problems are complex, and possible solutions are often burdened with technological barriers. As a last resort, overcoming the operational difficulties may even lead to changes in the construction of the WWTP technological system. The efficiency of the biological wastewater treatment process can be significantly improved by using the integrated fixed-film activated sludge (IFAS) technology, i.e., a system of suspended biomass in combination with biomass settled on a textile bed. Importantly, thanks to its modular structure, this technology can be used in existing facilities of various structures without the need to expand the system, making it a literally “tailor-made” solution.

**Figure 1:**
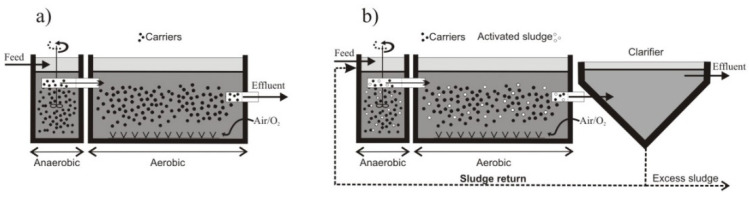
Schematic comparison of moving bed biofilm bioreactor (MBBR) (**a**) and integrated fixed-film activated sludge (IFAS) (**b**).

There are two alternative technological solutions that combine the operation of conventional activated sludge chambers and bio-beds. The IFAS and moving bed biofilm reactor (MBBR) technologies have been used since the 1990s with various modifications [10]. In particular, solutions have been implemented to improve the efficiency of removing nutrients such as total nitrogen (TN) and total phosphorus (TP), but also to improve sludge sedimentation properties and more efficiently reduce biological oxygen demand (BOD). Both technologies use bio-carriers in the form of fibers, materials, or bio-balls that increase the active surface inhabited by bacteria or other microorganisms of the activated sludge. The main difference between the solutions is that in the case of IFAS, some of the activated sludge is returned to the denitrification chamber (Figure 1b). The process of removing ammonium nitrogen (NH4-N) is carried out both on the carriers and on the surface of the flocs. In MBBR reactors, microorganisms forming the biofilm grow on the surface and inside the bio-carriers, and it is mainly them that take part in the wastewater treatment process (Figure 1a). There are currently many modifications of the IFAS system; typically, the carriers are placed only in the aerobic zone.

Research by Malovanyy et al. [11] demonstrates the higher efficiency of the IFAS reactor compared to the MBBR reactor. According to Ødegaard [10], nitrification in IFAS systems is achieved at lower retention times (RT) compared to the conventional system; lower RT results in a greater proportion of carbon in the denitrification processes. Denitrification in IFAS reactors can be performed as pre-, post-, and combined denitrification [10]. The results of research by Vergine et al. [12] indicate that even with full aeration, intensive denitrification can occur due to the fouling of the bio-carriers. On the other hand, phosphorus removal in reactors can be performed as chemical precipitation or using biological methods [13]. In the case of nitrification, Randall et al. [14] have demonstrated that this process can become more efficient by enriching the reactor with nitrifying microorganisms; however, the authors also emphasize that as chemical oxygen demand (COD) increases, competition between heterotrophic bacteria in the biofilm can occur, resulting in the deterioration of the process. According to Shao et al. [15], the optimal conditions for nitrification in the IFAS-SBR reactor are achieved when COD is 150 mg/dm^3^.

It has also been shown that to ensure the proper parameters of the IFAS process, it is necessary to control the production of extracellular polymeric substances (EPS) for both biofilm and activated sludge. Its amount depends on the type of bacteria (heterotrophs produce more of it) and affects the transfer of nutrients into the biofilm. Interestingly, research by Piechna et al. [16] indicates that the removal of organic pollutants is mainly performed by bacteria in the activated sludge and not by those forming a biofilm on the bio-carriers. In terms of dissolved oxygen (DO) demand, it appears that it is lower for IFAS systems compared to MBBR [17], although studies [18] have shown that a DO concentration of 4 mg/dm^3^ is required to comply with the norm for ammonium ion concentration in the outflow. These findings are in general agreement with [19], where DO concentrations for nitrification and denitrification efficiencies in the IFAS reactor were found to be 4.5 and 2.5 mg/dm^3^, respectively.

Research by Johnson et al. [20] highlights the benefits of using bio-carrier solutions in winter. The authors’ research confirms that at low temperatures, the efficiency of ammonium ion oxidation (fraction of NH_4_-N oxidation activity) is higher on the carriers compared to the suspended sediment.

The authors of this publication had the opportunity to conduct research during the period of developing and expanding biofilm on the textile bed and to observe how this technology works at a full-scale activated sludge WWTP. This paper presents selected research results from six months (184 days) of operation of a WWTP using the IFAS technology. Although there are various solutions concerning bio-carriers in IFAS systems, the most common biomass carriers include free-floating media with a large active surface whose density is close to water density. The presented IFAS system with biomass settled on textile beds is one of the least common solutions. So far, little attention has been paid to the environmental and operational factors affecting protozoan and small metazoan in the IFAS system.

## 2. Material and Methods

### 2.1. Activated Sludge System Characteristics

**Figure 2:**
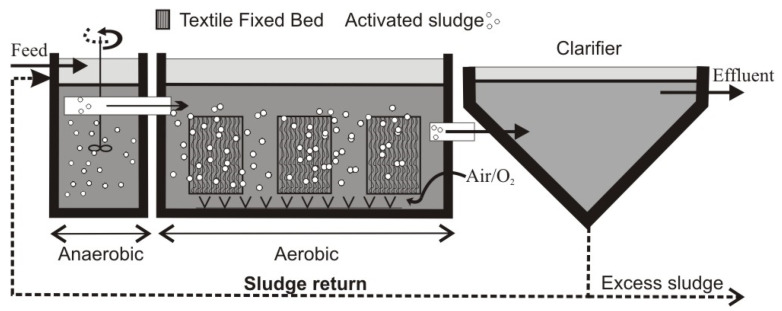
Simplified technological diagram of the WWTP in Mirków with the installed modules (textile fixed beds).

The system under study is a mechanical–biological WWTP in Mirków (Lower Silesia, Poland). Wastewater is delivered and transported to it via a combined sewer system whose maximum daily supply has been determined at 3700 m^3^/d and the maximum hourly supply at 280 m^3^/h. The treatment plant in Mirków is focused on the treatment of municipal wastewater using mechanical and biological methods with chemical precipitation of phosphorus. At the stage of mechanical wastewater treatment, debris and the mineral fraction are removed by the traveling screens and sand, respectively. The process of biological wastewater treatment begins in the chambers of the BIO I reactor. The reactor consists of eight chambers: an equalization tank, two dephosphatation chambers, three denitrification chambers, a stabilization tank, and a storage tank. Wastewater from the BIO I reactor flows to two BIO II reactors (marked as KN1 and KN2). In a single BIO II reactor, there are two separate chambers; the first can function as both a nitrification and denitrification chamber, while the second (main) chamber is solely a nitrification chamber (aerobic). Excess sludge from the BIO II reactor is recirculated to the first denitrification chamber in the BIO I reactor. After the treatment processes in the BIO II reactors, the wastewater from the nitrification chamber is transported through open channels to two radial secondary settling tanks (clarifiers), in which the sedimentation process occurs. From there, secondary sludge is returned to the pre-denitrification chamber or stabilization tank in the BIO I reactor. Figure 2 shows a simplified diagram presenting the technological system and modules.

### 2.2. IFAS System

**Figure 3:**
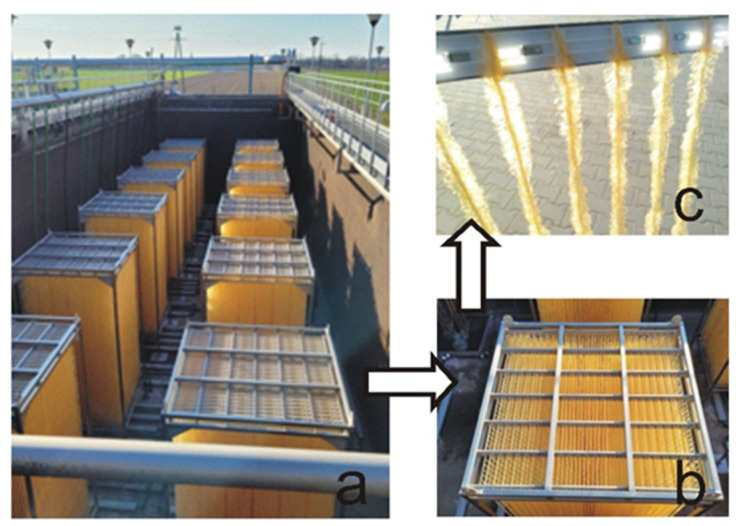
Cleartec BioCurlz biological bed modules (IFAS technology) attached to the activated sludge chambers (**a**); single module with biotextile for biofilm development (**b**); fragment of biotextile before installation–close-up (**c**).

Modernization work aimed at the implementation of the IFAS technology started at the treatment plant in January 2021, without additional changes to the existing technological system. Bio-carriers in the form of fibers grouped in modules were placed only in the aerobic zone of the KN1 and KN2 chambers. A total of 24 modules were installed, 12 in each chamber. The arrangement of modules during installation in the KN2 chamber is shown in Figure 3.

Installing bio-beds in the treatment plant was intended to solve problems connected with higher pollutant load in the KN1 and KN2 chambers by increasing the amount of biomass, which would improve the efficiency of wastewater treatment in the existing chambers. WWTP in Mirków for many years struggle with variable inflow and high pollutant load. The increase in biomass occurs through its growth on a textile bed made of a combination of polypropylene and saran (PVdC). Due to this, it is assumed that the biomass in the chambers will be concentrated in modules, thus creating the right conditions for processing an increased load of pollutants and treating more wastewater per time unit.

Bio-beds were initially installed in the KN1 chamber, while the KN2 chamber kept operating according to the traditional system. In March 2021, the modules were installed in the KN2 chamber, by which time the KN1 chamber had already been operating in the fixed and suspended biomass technology. Sewage was directed to the KN2 chamber in April 2021, and sampling began two weeks after launching its operation.

### 2.3. Analytical Methods

**Figure 4:**
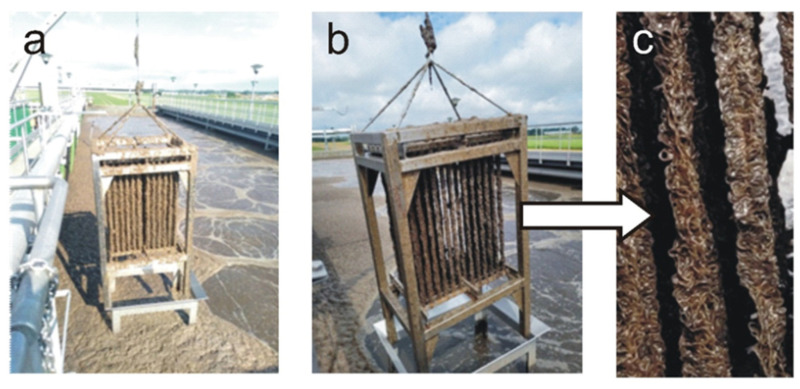
Single textile bed module for collecting sediment samples: fibers with a thick layer of sediment on the 59th day of operation (**a**); fibers with a thin sediment layer and visible clearances on the 114th day of operation (**b**); close-up of fibers on the 114th day of operation (**c**).

Samples for microbiological and qualitative analyses (sludge settleability, pollution indicators) were collected from April to October 2021, which corresponds to 184 days of operation. Activated sludge samples were collected only from the KN2 chamber in two forms: as suspended biomass (sludge from the sludge chamber) and as fixed biomass (sludge collected from a single fiber). Collecting sludge from the fibers was possible thanks to a small removable control module that could be pulled out through an opening in the KN2 chamber (Figure 4). Samples of treated wastewater for qualitative analyses were taken from a collecting well located behind the secondary settling tanks before the sewage was discharged into the receiving tank; raw sewage was collected at the inflow to the treatment plant.

Microbiological tests were carried out using confocal microscopy (Nikon Eclipse Ni-E C2, Japan) equipped with a 5-megapixel color digital camera (DS-Fi1c) and Nikon CFI 60 brightfield system. More than 20 random fields in each sample plate were viewed to determine the qualitative and semi-quantitative content of the biological component. The presence of protozoan and metazoan was marked by a numerical value. Calculation of % relative abundance was carried out by taking into account the total number of micro- and macrofauna and presence index (PI) (0.5—rare; 1—secondary; 1.5—super secondary; 3—dominant; 3.5—super dominant). To analyze the samples with the fluorescence in situ hybridization (FISH) method, Amann’s standard protocol was used [21]. The following hybridization probes were used: EUB338 (universal oligonucleotide probe) and NSO1225 for identifying AOB (ammonia-oxidizing β-proteobacteria) labeled with 6-carboxyfluorescein (6-FAM) dye. *Candidatus* Accumulibacter phosphatis was identified with the PAO462 probe labeled with (ROX) dye, and *Candidatus* Competibacter phosphatis was identified with the GAOQ431 probe labeled with (6-FAM) dye. 4′,6-diamidino-2-phenylindole (DAPI) was used to identify the DNA of all microorganisms in the sample.

The sludge volume index (SVI) test was carried out in a classic way in a 1 L measuring cylinder after settling the mixed liquor total suspended solids (MLTSS) for 30 min. The effectiveness of the treatment process was assessed, among others, by analyzing standard quality parameters, i.e., BOD, COD, total suspended solids (TSS), TN, and TP in wastewater. The same parameters were also analyzed in activated sludge samples obtained from the KN2 chamber in order to better identify the functioning of the system. Food-to-microorganism (F/M) from mixed liquor and BOD/COD ratios were determined using the value of mixed liquor volatile suspended solids (MLVSS). Table 1 shows the methods and standards for individual parameters for which pollution indicators were determined.

## 3. Results and Discussion

### 3.1. Activated Sludge Microorganisms Settled on Textile Beds

**Figure 5:**
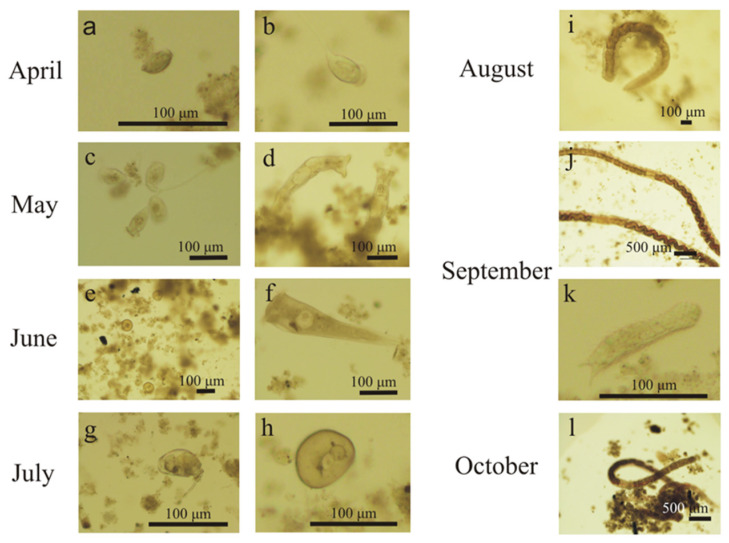
Examples of characteristic microorganisms observed on the fibers during 184 days of operation from April to October 2021 figures (**a**–**l**) are referred to in thet ext).

The most common organisms involved in the biodegradation of organic compounds are bacteria, protozoan, and small metazoan. Bacteria play a central role in the wastewater purification system; protozoan and small metazoan are also essential due to their grazing of a majority of bacteria. The microbiological composition, and in particular the dominance of any particular groups of microorganisms, may provide information about irregularities in the wastewater treatment process. Figure 5 shows the changes observed in the population of biofilm obtained directly from the fibers.

In April (1–16 days of operation), filamentous microorganisms, crawling ciliates (e.g., *Aspidisca* sp.) (Figure 5a), free-swimming ciliates (e.g., *Litonotus* sp.), and attached ciliates (e.g., *Vorticella* sp.) (Figure 5b) were observed on the fibers alongside multicellular organisms (nematodes). Protozoans play an important role in a biological reactor, and their primary function is to “rejuvenate” the activated sludge by removing dead organic matter and forming sludge flocs as a result of the production of extracellular substance [22]. One of the species that indicates good oxygenation is *Aspidisca costata* crawling ciliates, which was abundant in all the tested samples. On the 45th day of operation, attached ciliates with a more extensive structure were observed (Figure 5c); testate amoebae (mainly *Arcella* genus) and a large number of rotifers (Figure 5d) also appeared. Their presence indicates a stable and well-oxygenated sediment, characterized by low pollutant load and long age. However, a large number of rotifers may cause excessive sludge fragmentation, which lowers the quality of sewage at the outflow [23]. In June (day 59), in addition to the aforementioned microorganisms, very numerous amoebae (Figure 5e) and ciliates (*Stentor* sp.) were noticed (Figure 5f). Their presence is a positive phenomenon that proves that the wastewater has a low pollutant load and is well oxygenated. At that time, the sediment was characterized by a high species diversity (more than 10 taxa) without clear dominance of any one species. In July (day 88), numerous rotifers (Figure 5g) and amoebas of the *Arcella* genus (Figure 5h) were observed, while the presence of attached ciliates suggested a stable operation of the activated sludge, which was confirmed by the results of physico-chemical analyses. It would be alarming if the number of attached ciliates exceeded 80% or if the species *Opercularia* sp. and *Vorticella microstoma* appeared [23]. However, in the activated sludge samples collected from the fibers, no increase in the abundance of these species, or in the abundance of flagellates, was observed.

**Figure 6:**
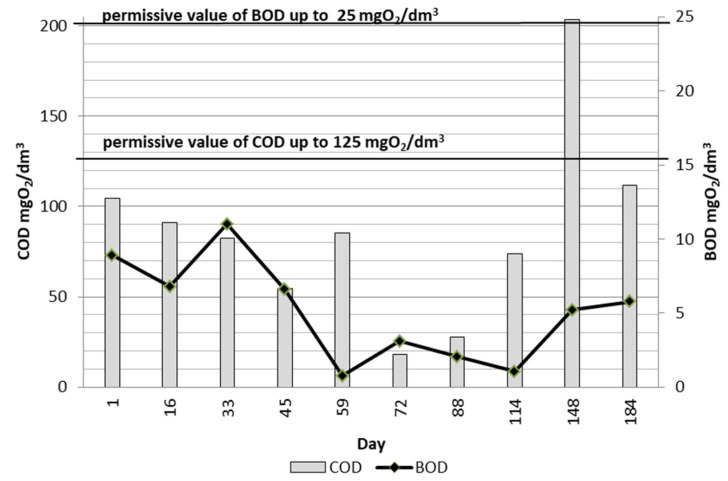
Changes in COD and BOD concentrations in treated wastewater from the 1st to the 184th day of operation (April–October 2021).

In August (114th day of operation), very numerous oligochaetes (Figure 5i) appeared and dominated the sediment obtained from the fibers. The appearance of the module changed significantly during this period. There was only a thin layer of sediment on the fibers, which was in great contrast to the initially thick fibers, densely covered with biomass (Figure 4). A similar situation was observed between the 148th and 184th day of operation (Figure 5j,l). The presence of *Spirogyra* sp., gastrotrichs (Figure 5k), and nematodes was also observed in the sediment. According to [22], an increase in the number of multicellular organisms results from nutrient deficiency and oxygen concentration. Since the oxygen level in the reactor was kept at the level of over 2 mg/dm^3^, the reason for the appearance of such a large group of oligochaetes was not connected with the DO concentration. The analysis of physicochemical parameters showed that COD at the outflow significantly increased in September (Figure 6), which coincided with the appearance of oligochaetes. Elevated concentrations of COD in the reactor, with values exceeding 380 mg/dm^3^, had already been found in August.

**Figure 7:**
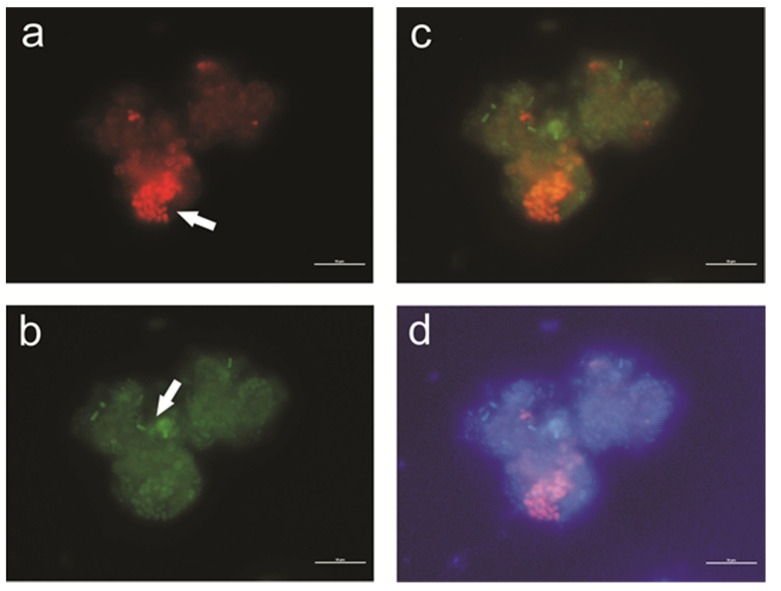
FISH hybridization of bacteria from the IFAS reactor. *Candidatus* Accumulibacter phosphatis identified with the PAO462 probe (**a**) and *Candidatus* Competibacter phosphatis identified with the GAOQ431 probe (**b**) in activated sludge. The effect of overlaying images a + b (**c**), a + b + c (**d**), and a + b + DAPI (**d**). Scale bar: 10 μm.

**Figure 8:**
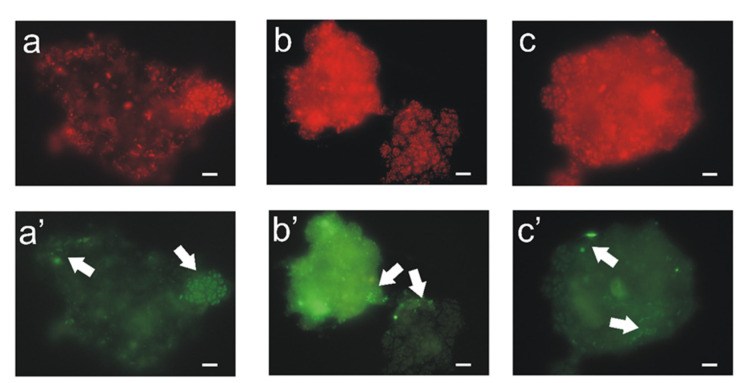
Fish hybridization of bacteria from the IFAS reactor. Most bacteria identified with the EUB338 probe (**a**–**c**) and ammonia-oxidizing β-proteobacteria (**a’**–**c’**) in activated sludge (**a**,**a’**), in biofilm from the outer surface of the biotextile (**b**,**b’**), and at the outflow (**c**,**c’**). Scale bar: 10 μm.

In addition to the growth of micro- and macrofauna, bacteria also developed on the bio-beds. The bacterial diversity of a WWTP with IFAS system has been presented by Kwon [24]. The structure of the modules enables the creation of anaerobic, aerobic, and anoxic zones within them, which can be inhabited by both nitrifying bacteria and denitrifying bacteria, those accumulating phosphorus (PAO) and glycogen (GAO). Figure 7 shows *Candidatus* Accumulibacter phosphatis and *Candidatus* Competibacter phosphatis on a single activated sludge floc, with a predominance of phosphorus-accumulating bacteria. The arrangement of fibers within the modules and the formation of biofilm on them leads to the simultaneous occurrence of all processes involved in the removal of biogenic compounds within the activated sludge chamber. Importantly, the first-step nitrification bacteria were also found on the fibers. The application of the FISH method and the NSO1225 probe made it possible to identify these bacteria (ammonia-oxidizing β-proteobacteria). The tests were performed in April (day 1), May (day 33), August (day 114), and September (day 148). Bacteria of the first step of nitrification were observed both on the fibers and in the activated sludge. Additionally, examination of treated wastewater indicated the presence of these bacteria also at the outflow (Figure 8c’). A similar observation is made in [25], where a large fraction of the AOB community was also present in the bulk water. Importantly, research by Suarez [25] shows that the bacteria inhabiting textile beds are not specially protected from predation, i.e., amoeba grazing.

### 3.2. Activated Suspended Sludge Microorganisms in the Bulk Water

**Figure 9:**
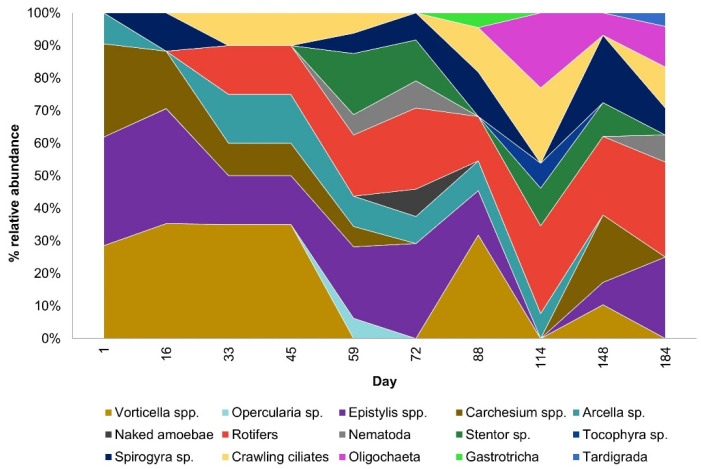
Composition of protozoan and metazoan in activated sludge samples during 184 days of operation.

**Figure 10:**
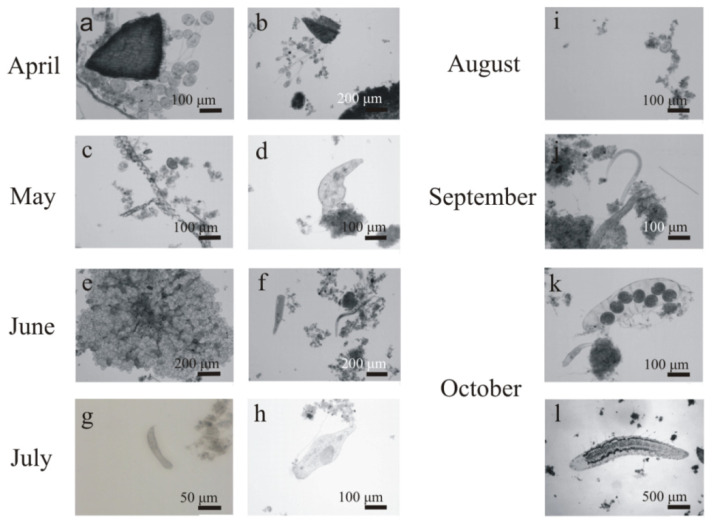
Examples of characteristic microorganisms observed in the activated sludge during the period of 184 days of operation from April to October 2021 (figures (**a**–**l**) are referred to in the text).

The changes observed in the microbial composition of the activated sludge coincided with the changes occurring on the fibers. The composition of the sludge flocs included bacteria as well as protozoan and metazoan communities. The occurrence of protozoan and metazoan in activated sludge samples is shown in Figure 9. Examples of characteristic microorganisms observed in the activated sludge are shown in Figure 10.

The composition of activated sludge during 184 days of operation reflected the differences in the microbial community composition. The ongoing recomposition of the microfauna resulted in the changing dominance of individual species. The observed changes are in agreement with the common knowledge that protozoans, especially ciliates, are generally the predominant species [26]. According to [26,27], 70% of the protozoan species in sludge are ciliates. The presence of ciliates is typically found in young to medium age sludge [27], but they were present throughout the research period. The largest percentage of relative abundance of ciliates (*Vorticella* spp., *Epistylis* spp., *Carchesium* spp., *Arcella* sp., and *Spirogyra* sp.) occurred between the 1st and 33rd day of operation (Figure 9). Metazoan, divided into rotifers, oligochaetes, nematodes, tardigrades, and gastrotrichs, played a secondary role, but after the 59th day of operation, their relative abundance increased (Figure 9). The aeration tank operated over 2 mg/dm^3^; greater availability of DO results in the development of the metazoan community. Numerous microorganisms were found in the sediment, attesting to its low BOD load. Rotifers (Figure 10d,h), nematodes (Figure 10j), testate amoebas (Figure 10i), crawling ciliates, and attached ciliates of the *Carchesium* sp. (Figure 10a) are precursors of sediment with a low pollutant load, good oxygen conditions, and longer sludge age. A low load combined with high oxygen content resulted in the appearance of *Stentor* sp. (Figure 10f), oligochaetes (Figure 10l), nematodes, gastrotrichs (Figure 10g), and even tardigrades (<0.1 BOD) (Figure 10k) [28].

**Figure 11:**
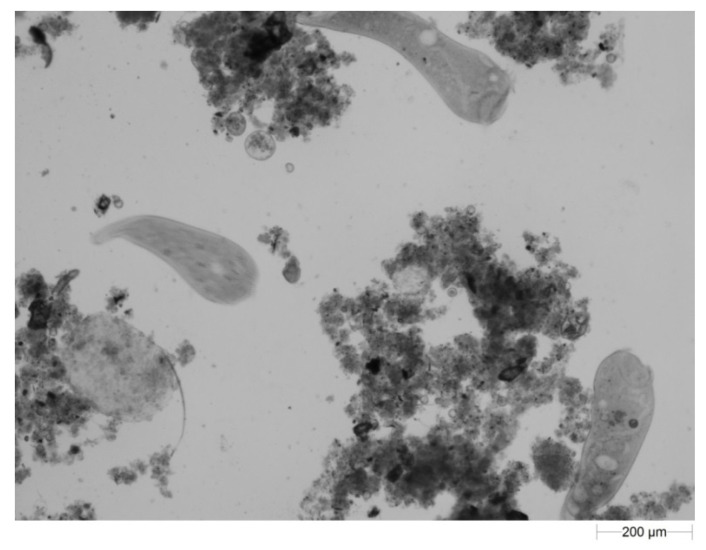
*Stentor* sp. in activated sludge—59th day of operation.

A particularly interesting discovery was the appearance of large numbers of *Stentor* sp. that usually inhabits freshwater environments [29,30], but this protozoan was actually the second most common microorganism in activated sludge between the 33rd and 72nd day of operation (Figure 11). Its abundance decreased after day 72, but it was still present in the sediment and on beds. The shape of *Stentor* sp. is highly variable due to the contractility of its body. Consequently, it is difficult to distinguish between the different species as they all look alike in a shrunken form. The shape also depends on whether the cell is attached or moves freely [31]. There are few publications on their preferences concerning the optimal conditions occurring in WWTP environments. However, they are known to breed easily; their grazing on bacteria usually indicates a stable wastewater environment and healthy biomass. According to Martín-Cereceda et al. [31], *Stentor coreuleus* is rare because it is sensitive to negative environmental factors. Therefore, it is worth considering whether their strong presence in the treatment plant was related to the use of the IFAS technology or it was due to other factors.

What was also discovered is that potentially pathogenic bacteria were preferentially associated with *Stentor* sp. cells [32]. Pucciarelli et al. [33] indicate that their sudden bloom in fresh waters may be connected with large fluctuations in water temperature. In this case, the average temperature in the period of their intense occurrence in April (day 59) was 12 °C, in May (day 72)—15 °C, and in June (day 88)—already 22 °C. Moreover, their appearance in the activated sludge chambers coincided with the detection of high concentrations of total nitrogen. Importantly, Klimek et al. [34] indicate that *Stentor* sp. is sensitive to the ammonia concentration. During the period of their appearance in the activated sludge chambers, no high concentrations of ammonium nitrogen were found. The ammonia concentration in activated sludge was 0.3 mg/dm^3^, 0.31 mg/dm^3^ and 0.15 mg/dm^3^.

According to [35], various species of *Stentor* spp. have different oxygen requirements. Some species live at the bottom of tanks where anaerobic conditions predominate, while others appear at the surface, where there is greater availability of oxygen. With low oxygen levels, digestion is inhibited, and *Stentor* spp. becomes stuffed with undigested food vacuoles. In the reactor, *Stentor* spp. has the ability to live both in aerobic conditions and under oxidation (fibers, biofilm on a metal structure). Research by González-Pleiter et al. [36] seems particularly interesting in the context of the presence of *Stentor* sp. The authors conducted an experiment on microbial colonizers of non-biodegradable and biodegradable microplastics (MP) with both spiky and soft surfaces. They were put into metallic cages and deployed for eleven days into a lake. It turned out that MPs were characterized by a significantly high relative abundance of ciliates such as *Stentor* sp. The species was clearly dominant in both non-biodegradable (53.8%) as well as biodegradable MPs (30.2%). This could suggest that in combination with good aerobic conditions, artificial Cleartec BioCurlz fibers create the right environment for the development of *Stentor* sp. As this species grew on the fibers, its amount in the suspended sediment increased. It seems natural since the surface of the attached biomass has contact with the suspended biomass in the bioreactor.

### 3.3. Treatment Efficiency

The composition of the feed wastewater based on the 10 measurements is as follows. COD values varied from 760 to 1440 mg/dm^3^ and BOD from 320 to 690 mg/dm^3^ with a median of 949 and 475 mg/dm^3^, respectively. The concentrations of TN oscillated from 84 to 140 mg/dm^3^ and TP from 8.3 to 21 mg/dm^3^ with a median of 110 and 13 mg/dm^3^, respectively. The highest values of COD and BOD occurred in May 2021, whereas the highest concentrations of TN and TP were observed in September 2021. Ammonia nitrogen concentrations at the inflow varied from 61 to 89 mg/dm^3^ (with a median of 89 mg/dm^3^). TSS changed from 140 to 826 mg/dm^3^, while its elevated concentrations, with values exceeding 720 mg/dm^3^, had already been found in April and May.

**Figure 12:**
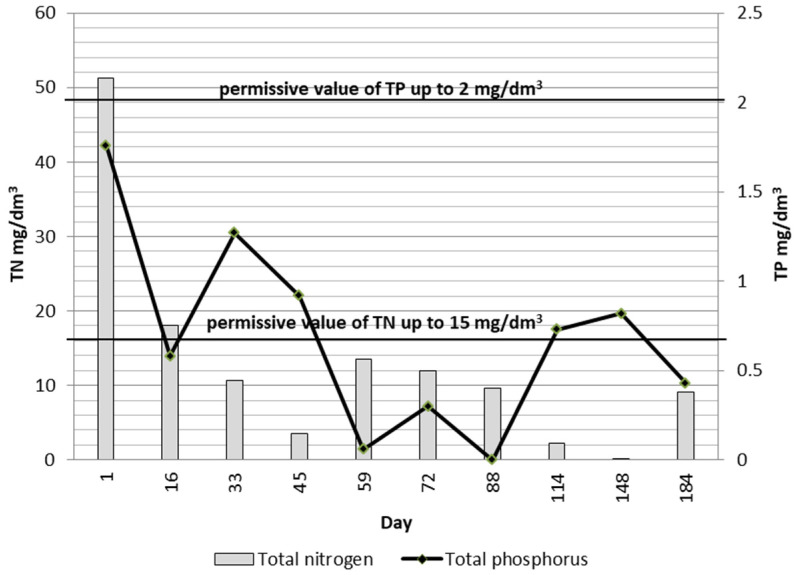
Changes in N and P concentrations in treated wastewater from the 1st to the 184th day of operation (April–October 2021).

**Figure 13:**
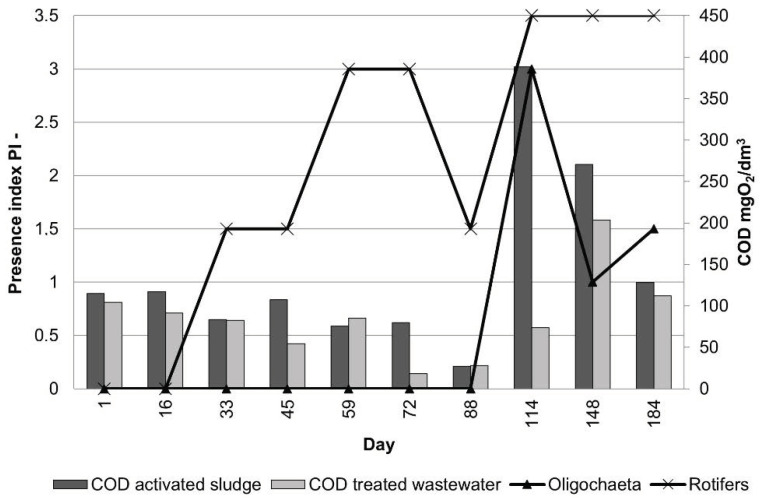
Changes in COD concentrations in treated wastewater from the 1st to the 184th day of operation (April–October 2021) with the Oligochaeta and rotifers presence index.

Figure 6 and Figure 12 show the distribution of the analyzed parameters of treated wastewater. The test results confirm the effectiveness of the treatment process, although samples of treated wastewater obtained in the initial period of operation from the KN2 chamber (day 16) contained the highest TN concentrations in the entire study period (51.31 mg N/dm^3^ and 18.06 mg N/dm^3^), whereas later on the typical values were below 15 mg N/dm^3^. At this time, the concentrations of BOD, COD, and TP were also above the six-month average. BOD concentrations were in the range of 0.76–11 mg O_2_/dm^3^ with an average of 5.12 mg O_2_/dm^3^, which means that they were within the permissible values for treated sewage. The content of TP in the treated wastewater, with an average of 0.67 mg P/dm^3^, was within the standard range throughout the entire study period. In the case of COD, only the sample collected on the 148th day of operation showed an increased concentration of 203.6 mg O_2_/dm^3^; on other days, the COD concentration values did not exceed the acceptable standard, although the COD value increased. TSS values often exceeded the norms. It may have been caused by metazoan grazers, whose excessive abundance was detected after the 59th day of operation. Metazoans break up the biological mass, which causes an increase in suspension at the outflow [6]. Figure 13 presents COD in treated wastewater, activated sludge, and PI values. There is a relation between the increase in COD and the excessive appearance of oligochaetes and rotifers. It is noteworthy, however, that other authors [26,37] did not find significant relationships between specific taxa and the quality of the effluent in terms of BOD or COD. Fazelipour et al. [38] also found no correlation between nitrogen concentration in the IFAS-OSA bioreactors and the changes in the number of protozoan and metazoan communities. The presented research data at this point may not sufficiently explain the relation between protozoan and metazoan inhabiting activated sludge and COD in wastewater. The research is still ongoing, which may provide further valuable information to find a statistical correlation. However, the presence of microorganisms in the activated sludge may be an indicator of effluent quality. One of such established indicators is the presence of tardigrades and *Stentor* spp., which only occur in wastewater of very good quality [39].

**Figure 14:**
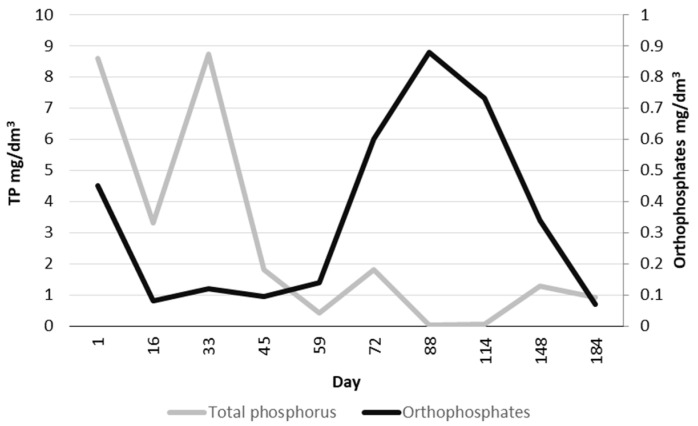
Changes in the concentration of TP and orthophosphates in the activated sludge.

When analyzing TP during the first two months, first, a clear decrease was observed, and in the following months, the share of orthophosphates increased (Figure 14). Klimiuk and Łebkowska [40] report that orthophosphates constitute 50–70% of TP. Removal of orthophosphates with lime is effective, while in order to remove organic phosphorus, the expected results are achieved by using iron (III) sulfate (VI) called PIX. In the Mirków WWTP, chemical phosphorus precipitation with PIX is used. Phosphorus compounds in wastewater occur in an inorganic form, as polyphosphates and orthophosphates, as well as in an organic form. In biological anaerobic processes, orthophosphates are released from wastewater. In order to eliminate phosphates from wastewater, denitrification dephosphatation is also used, which involves bacteria that accumulate phosphates under anaerobic conditions. This process also reduces nitrates, but it is less effective than traditional denitrification and requires more knowledge. According to research by Hu et al. [41], the elongation of anaerobic phases in the IFAS-MBSBBR reactor increases the enrichment of biomass in PAO representatives and improves nutrient removal efficiency. In the conducted research, the presence of phosphorus-accumulating bacteria was observed (Figure 7), although additional tests are needed to confirm their involvement in the phosphorus removal process. Nevertheless, due to the use of textile modules, these bacteria have access to both nitrates formed in the aerobic phase (ammonia oxidation) and orthophosphates, which penetrate the layers of biomass formed on the fibers [42].

The detection of relatively few elevated concentration values for the analyzed parameters may have resulted from the changes occurring in the activated sludge chamber. It should be stressed that the process of biofilm growth on textile beds was just beginning in the research period, while the suspended biomass was developing and adapting to the conditions in the chambers. The concentration of dissolved oxygen was maintained over 2 mg O_2_/dm^3^, and the concentration of suspended biomass in the KN2 chamber varied significantly from 3.37 g/dm^3^ to as much as 9.98 g/dm^3^. Such high biomass concentration can be explained by the changes in the textile beds during operation. A natural phenomenon occurring in the system is that a certain part of the biofilm becomes gradually detached from the beds and moves into the activated sludge chamber, thus forming the suspended biomass. In the initial period of research and operation of the beds, the cyclicality and scale of this phenomenon could not be established. Correct determination of the sludge sediment suspended on the fibers is still a challenge.

The development of the sewage infrastructure influenced the constantly increasing wastewater inflow. For this reason, the nitrification/denitrification process is still being optimized. The solids retention time (SRT) is about 10 h and varies depending on the load of the chambers.

### 3.4. Wastewater Control Information

In activated sludge systems, the food to microorganism (F/M) ratio is a key operational factor. There must be a balance between the food (entering the bioreactor) and the microorganisms (in the bioreactor). Liang et al. [43] indicate that higher growth rates of oligochaetes are observed in sludge with higher protein content and when the F/M ratio is lower than 0.7 (mg COD/mg VSS day). Free swimming ciliates, in turn, are usually associated with high F/M (mg COD/mg VSS day) ratios [44]. These findings are in good agreement with our research. F/M ratios fluctuated from 1.307 to 0.287 (mg COD/mg MLVSS day), and after the 59th day of operation, they never exceeded 0.7 (Table 2). The obtained value corresponds with limited growth of microorganisms (endogenous phase) and high predatory activity. The optimal range for the F/M ratio in a complete mix bioreactor is from 0.2 to 0.6 (kg BOD/kg MLVSS day), but it is preferred when the value is closer to the upper limit. A low F/M ratio occurred throughout the research period, which means that the activated sludge process can be better optimized.

The COD/BOD ratio in the wastewater flowing into the treatment plant is a measure of the biological distribution of pollutants. At a ratio of less than 2, good biodegradation efficiency is observed. In the analyzed samples, the ratio ranged from 3.093 to 1.750 throughout the study period. The changes in the COD/BOD ratio were not actually reflected in the changes in the activated sludge environment. The COD/N ratio is also correlated with the treatment process efficiency. According to Fu et al. [45], TN removal efficiency decreased as the COD/N ratio dropped from 9.3 to 5.3. In the analyzed samples, this ratio ranged from 11.809 to 13.091 from the 1st to the 45th day of operation. After day 59, this parameter began to drop, eventually reaching 7.786 (day 148). This coincided with the appearance of excessive amounts of small metazoan in the activated sludge and fiber biofilm samples. The TN removal efficiency did not decline.

### 3.5. Activated Sludge Settleability

**Figure 15:**
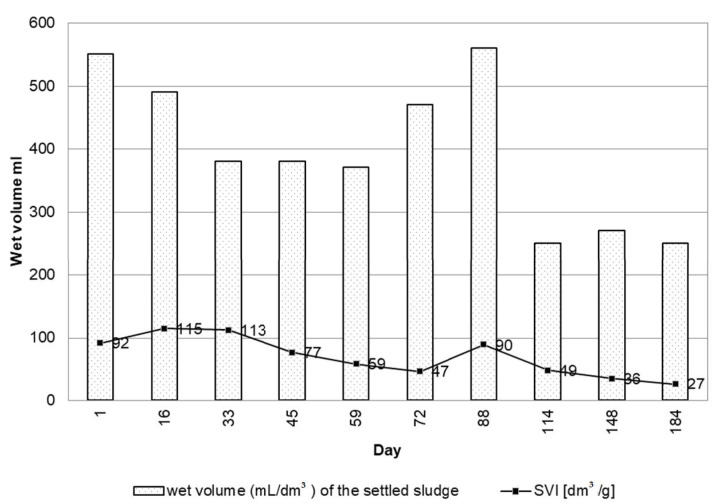
Results of sedimentation tests conducted from the 1st to 184th day of operation (April–October 2021).

Ødegaard et al. [10] emphasize that IFAS systems improve the sedimentation properties of sludge. During the research period, the suspended sediment had a brown color and an earthy odor. It settled rather well in the measuring cylinder. On days 72 and 88, the volume of the settled sludge was high, which corresponded with high biomass concentrations obtained on these days (9.98 g/dm^3^ and 6.25 g/dm^3^). The results of sedimentation tests with the calculated sludge index are presented in Figure 15. Periodically high biomass concentrations in the activated sludge chamber did not negatively influence the quality of sedimentation. According to [19], high DO concentration has a positive effect on sludge settleability and SVI values. It has also been established that oligochaetes can be beneficial as they stabilize the sludge settleability [43]. Additionally, the occurrence and density of metazoan, especially rotifers, have been found to be correlated with SVI [46]. In this case, the period of the presence of oligochaetes and the dominance of rotifers also coincided with better sedimentation (day 114).

## 4. Conclusions

A novel aspect of this study is the investigation of the impact of the IFAS system on activated sludge biocenosis, which has not been performed before so precisely with respect to protozoan and small metazoan. To date, little attention has been paid to microbial communities and operational factors in the IFAS system. This study is a fairly comprehensive examination of the operation of a WWTP during the period of six months after the implementation of IFAS. The results obtained in this study focused on microbial diversity and its influence on treatment effectiveness rather than the quantitative distribution of specific species. Using morphological analysis, it was possible to characterize protozoan and metazoan involved in the wastewater treatment process.

In summary, the compositions and dynamics of protozoan and small metazoan affected the efficiency and stability of a full-scale IFAS system. A high content of DO (over 2 mg/dm^3^) and F/M ratio above 0.7 (mg COD/mg MLVSS) create favorable conditions for the development of protozoa in suspended and attached biomass. In the case of *Stentor* sp., it is presumed that its increased occurrence may be caused by both favorable oxygen conditions and a low concentration of ammonium ions with a generally low F/M ratios (both COD and BOD).

During the entire six-month observation period, the textile beds were covered with microbiologically active biofilm. However, after day 72, the process seemed to break down. Excessive occurrence of metazoan led to reduced sludge production and a significantly decreased rate of biofilm formation. Low F/M ratios and increased COD in the activated sludge and treated wastewater coincided with the appearance of oligochaetes and rotifers, which partially contributed to sludge fragmentation and a greater amount of suspended biomass at the outflow. The presence of phosphorus-accumulating bacteria can reduce the demand for phosphorus-precipitating chemicals. However, additional research into biological dephosphatation is needed.

In general, using the IFAS technology positively affects the growth of the activated sludge microflora. In order to fully evaluate the operation of the reactor, further observations should be made during winter and spring, when the efficiency of wastewater treatment is most often reduced. The conducted tests indicate that the sludge parameters may decrease during the start-up.

## Figures and Tables

**Table 1:** Norms and methods for indicating pollution.

Pollution Indicator	Research Methodology	Norm
BOD	Specific method	PN-EN 1899-1:2002
COD	Specific method	PN-ISO 6060:2006
TSS, MLTSS, MLVSS	General method	PN-EN 872:2002
TN	Calculation method	-
TP and orthophosphates	Spectrophotometric method with ammonium molybdate	PN-EN ISO 6878:2006

**Table 2:** F/M, COD/BOD, and COD/N ratios.

Day of Operation	MLVSS mg/dm^3^	F/M mg BOD/mg MLVSS	F/M mg COD/mg MLVSS	COD/BOD mg BOD/mg COD	COD/N mg BOD/mg TN
**1**	4293.36	0.257	0.796	3.093	11.809
**16**	3064.32	0.427	No data	No data	No data
**33**	2426.40	0.581	1.307	2.250	10.500
**45**	3543.84	0.490	1.023	2.087	13.091
**59**	4502.16	0.257	0.450	1.750	8.050
**72**	7185.60	0.116	0.332	2.876	8.627
**88**	4502.16	0.274	0.503	1.835	10.702
**114**	3682.80	0.287	0.580	2.019	8.742
**148**	5381.28	0.262	0.510	1.946	7.786
**184**	6677.28	0.121	0.287	2.375	8.000

## Data Availability

Not applicable.
